# Clinical Features of Painful Ophthalmoplegia with a High-Intensity Ring Appearance around the Optic Nerve on MRI: A Case Series

**DOI:** 10.1155/2020/6737018

**Published:** 2020-03-30

**Authors:** Yasunobu Nosaki, Ken Ohyama, Maki Watanabe, Takamasa Yokoi, Katsushige Iwai

**Affiliations:** Department of Neurology, Toyohashi Municipal Hospital, Toyohashi, Japan

## Abstract

**Objective:**

Painful ophthalmoplegia includes nonspecific magnetic resonance imaging (MRI) manifestations and various clinical features including orbital pain and cranial nerve palsies. Treatment for painful ophthalmoplegia remains controversial. The aim of this report was to describe detailed clinical features, MRI findings, treatments, and prognosis of patients with painful ophthalmoplegia. *Patients and Methods*. We retrospectively investigated four cases of patients with painful ophthalmoplegia diagnosed using the International Classification of Headache Disorders, 3rd edition.

**Results:**

All patients experienced unilateral orbital pain and oculomotor nerve palsy with diplopia but no vision loss. One of the four patients was diagnosed with Tolosa–Hunt syndrome based on the appearance of a granulomatous inflammation of the cavernous sinus on MRI. No specific lesions were detected on brain MRI for the other three patients; therefore, their headaches were attributed to ischaemic ocular motor nerve palsy. In all patients, a high-intensity ring appearance around the ipsilateral optic nerve was observed on MRI. Steroid therapy was administered to these patients, and good prognoses were anticipated.

**Conclusion:**

These results indicate that prednisolone is a useful treatment for painful ophthalmoplegia that displays ipsilateral hyperintense ring lesions around the optic nerve on MRI, regardless of the presence of granulomatous inflammation of the cavernous sinus.

## 1. Introduction

Painful ophthalmoplegia is a pathologic condition caused by nonspecific inflammation of the cavernous sinus due to tumors, vasculitis, basal meningitis, neurosarcoidosis, or diabetes [1]. It consists of periorbital or hemicranial pain with ipsilateral ocular motor nerve palsy and other cranial nerve palsies [1]. Due to the nonspecific nature of the inflammation, strategies for diagnosis, classifications, and treatment for painful ophthalmoplegia remain controversial. The International Classification of Headache Disorders, 3rd edition (ICHD-3), published in 2018, describes the diagnostic criteria of diseases applicable to painful ophthalmoplegia, including Tolosa–Hunt syndrome (THS), headache attributed to ischaemic ocular motor nerve palsy, and recurrent painful ophthalmoplegic neuropathy [2]. Brain magnetic resonance imaging (MRI) manifestations of painful ophthalmoplegia frequently reveal nonspecific findings, whereas some cases with normal brain MRI findings have been reported [3]. Herein, we present the cases of four patients with painful ophthalmoplegia diagnosed using ICHD-3 and describe the clinical features, MRI findings, and prognosis following considerable treatment.

## 2. Patients and Methods

**Figure 1:**
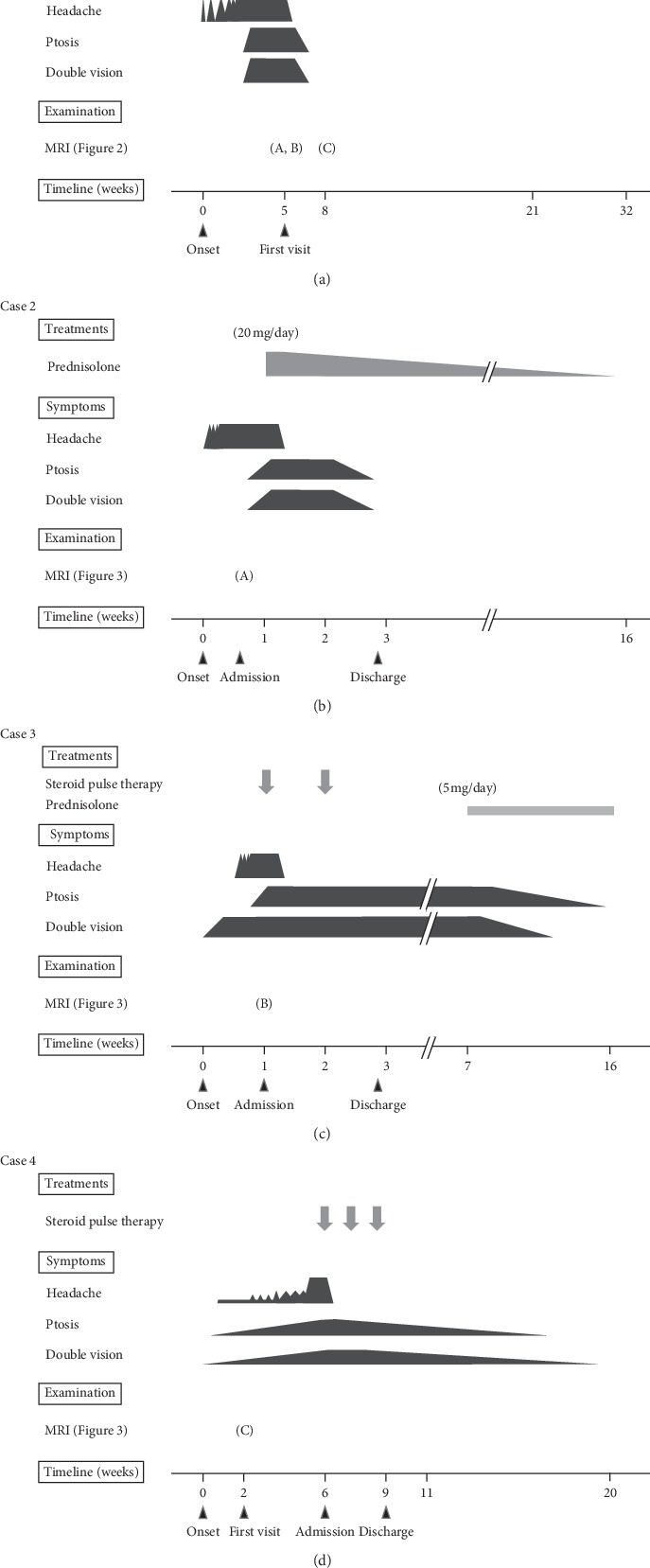
Clinical courses of patients with painful ophthalmoplegia during steroid therapy.

We retrospectively investigated four patients with painful ophthalmoplegia who were referred to the Toyohashi Municipal Hospital between October 2014 and April 2019. The diagnosis of painful ophthalmoplegia was based on the ICHD-3 criteria. All patients underwent clinical and neurologic assessments, brain MRI, and treatment for painful ophthalmoplegia. Patient characteristics and clinical courses are described below and summarized in Table 1 and Figure 1.

## 3. Report of Cases

### 3.1. Case 1

A 39-year-old man was referred for diplopia and left ptosis with severe headache persisting five weeks prior to the visit. Initially, he experienced recurring, abrupt, throbbing pain around his left cheek. The pain became more frequent and acquired a stabbing quality in the left periorbital and retro-orbital regions. Two weeks following onset of left unilateral pain, he developed left ptosis and his diplopia worsened with leftward gaze. At the time of the first visit to our department, he was alert, well-oriented, and afebrile. Although the neurological examination revealed third and sixth cranial nerve palsies, there were no visual field defects or visual disturbances. Conjunctival injection, chemosis, and periorbital edema were not observed in either eye. Funduscopic examination revealed no abnormalities in either eye, including the optic nerves. There were no neurological defects in other cranial nerve functions, the trunk of the patient, or his extremities.

**Figure 2:**
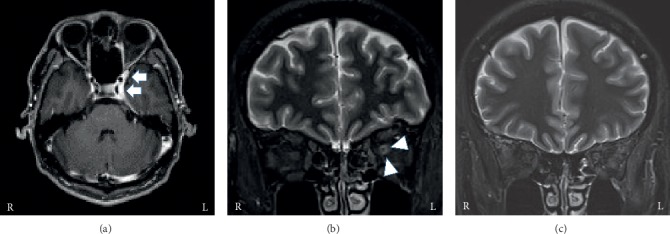
.Brain MRI of case 1. (a) The lesion (arrows) is enhanced on T1-weighted image and extends into the superior orbital fissure. (b) The high-intensity ring appearance (arrowheads) around the left optic nerve is seen on short tau inversion recovery (STIR) MRI. (c) The high-intensity ring appearance has disappeared on day 21 after initiating treatment.

Blood cell counts and blood chemistry tests were unremarkable. His C-reactive protein and *β*-D-glucan levels were normal. Tests for antineutrophil cytoplasmic autoantibody, myeloperoxidase antineutrophil cytoplasmic autoantibody, and Aspergillus and Cryptococcus antigens were negative. His brain MRI showed a mass lesion in the left cavernous sinus. The lesion was enhanced on the T1-weighted image and was observed to have extended into the superior orbital fissure (Figure 2(a)). Moreover, a high-intensity ring appearance around the left optic nerve was observed on short tau inversion recovery (STIR) MRI (Figure 2(b)).

This patient was diagnosed with THS and was treated with prednisolone (30 mg/day). Four days after treatment initiation, his headache improved. His diplopia and ptosis improved gradually after one week of steroid therapy. The patient fully recovered after oral prednisolone treatment which was subsequently tapered off over six months.

His follow-up brain MRI three weeks after starting treatment revealed a reduction of the asymmetrical high-intensity ring appearance around the optic nerve (Figure 2(c)). He experienced no recurrence of his symptoms after discontinuing prednisolone although MRI images indicated that the lesion in the cavernous sinus remained until eight months after the onset.

### 3.2. Case 2

A 68-year-old woman was admitted to our hospital with severe headache. Her headache had emerged successively from three days prior and deteriorated gradually. She felt persistent pinprick retro-orbital pain on the left side. On day two of hospitalization, diplopia developed. She had a history of non-insulin-dependent diabetes mellitus with preproliferative diabetic retinopathy and had been treated with metformin, pioglitazone, and glimepiride for 20 years.

She was alert and well-oriented. Her pupils and light reflex were normal, and ptosis was not observed. She presented with oculomotor palsy with limited adduction ability on the left side, indicating incomplete left cranial nerve III palsy. No other neurological abnormalities or visual disturbances were observed. Conjunctival injection, chemosis, and periorbital edema were not observed upon ophthalmological examination. Funduscopic examination revealed preproliferative diabetic retinopathy in both eyes. Central critical flicker fusion frequency (CFF) was 38 Hz in the right eye and 36 Hz in the left eye (reference >35 Hz).

**Figure 3:**
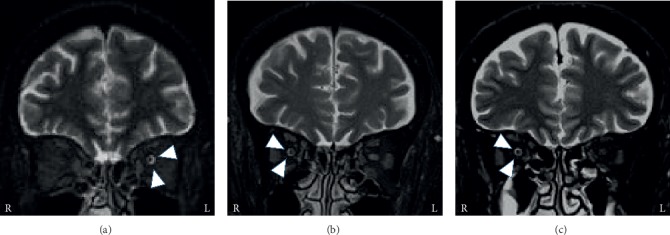
Brain MRI of cases 2–4. In case 2 (a), the high-intensity ring appearance (arrowheads) around the left optic nerve is found on STIR MRI. In cases 3 (b) and 4 (c), the high-intensity ring appearance (arrowheads) around the right optic nerve is found on fat-suppressed T2-weighted MRI.

Her laboratory examinations, including cerebrospinal fluid (CSF) study, were unremarkable other than hemoglobin A1c (HbA1c) level of 7.6% (reference, 4.6–6.2%). Her MRI revealed no abnormalities in the brain and cavernous sinus, while a high-intensity ring appearance around the left optic nerve was observed on STIR MRI (Figure 3(a)). Using ICHD-3, we diagnosed her with headache attributed to ischaemic ocular motor nerve palsy due to diabetes mellitus. An effective treatment for painful ophthalmoplegia in diabetes mellitus has not been determined. As we considered the existence of inflammation around the orbital lesion to be due to the high-intensity ring appearance on MRI, prednisolone was administered, in a manner similar to the management of THS. Two days after initiation of prednisolone (20 mg/day) her headache gradually improved. She was discharged with improvement of diplopia 10 days after admission. Prednisolone was tapered and discontinued after four months, and she remained symptom-free for four years.

### 3.3. Case 3

A 76-year-old man was admitted to our hospital with diplopia, right ptosis, and right retro-orbital severe pain which began one week prior to the visit. Initially, he experienced diplopia without pain. Three days later, he developed persistent throbbing pain around the right periorbital and retro-orbital regions. Six days after the onset of diplopia, he developed right ptosis. He had a history of hypertension, controlled with medication, and left branch retinal vein occlusion at 73 years of age.

At the time of visit, he was alert and well-oriented. His pupils and photopupillary reflex were normal, but ptosis was observed on the right side, indicating right cranial nerve III palsy. There were no other neurological abnormalities or visual field defects. Although he had slight visual disturbances in the left eye because of previous left retinal vein occlusion, he had no visual disturbances in the right eye. No abnormalities were observed in either eye upon further ophthalmological examinations, with the exception of left retinal branch vein occlusion.

Blood cell counts and blood chemistry tests were unremarkable. Although no abnormalities or mass lesions in the cavernous and paranasal sinus were observed in MRI, a high-intensity ring appearance around the right optic nerve was observed in fat-suppressed T2-weighted MRI (Figure 3(b)).

He was diagnosed with headache attributed to ischaemic ocular motor nerve palsy based on ICHD-3 due to risk factors for microvascular ischemia. As we considered the existence of inflammation around the orbital lesion to be due to the high-intensity ring appearance on MRI, prednisolone was administered, in a manner similar to the management of THS. On the following initiation of steroid pulse therapy (intravenous methylprednisolone 1000 mg/day for three days), his headache improved. A second steroid pulse therapy was initiated one week later due to gradual improvements in diplopia and right ptosis in response to prednisolone. He was discharged on day 18. Because of persistent ptosis, oral prednisolone (5 mg/day) was prescribed. His symptoms fully recovered after four months; therefore, prednisolone was discontinued. There was no recurrence of his symptoms for over six months.

### 3.4. Case 4

A 67-year-old man with a history of mild hypertension was referred to our hospital because of headache, right ptosis, and diplopia. He noticed diplopia 11 days before his first visit. Three days after developing diplopia, he experienced right ptosis and dull pain in the right periorbital region. He was admitted one month after his first visit because his headache, ptosis, and diplopia progressively deteriorated. On admission, he complained of continuous severe headaches in the right periorbital area. He was alert and well-oriented. The diameter of his pupil was 4 mm in the right eye and 2 mm in the left eye in a bright room. Pupil responses to light were dull in the right eye. Divergent squint and outward and downward displacement of the right eye were observed. No abnormalities in the visual field or visual acuity were observed. The conjunctival injection, chemosis, and periorbital edema were not detected upon an ophthalmological examination. Funduscopic examination revealed no abnormalities in either eye, including the optic nerves. Further, no neurological abnormalities of other cranial nerve functions, the trunk of the patient, and his extremities were observed.

Blood cell counts, blood chemistry, and cerebrospinal fluid tests were unremarkable. While no mass lesion in the cavernous sinus was detected by brain MRI, a high-intensity ring appearance around the right optic nerve was observed in fat-suppressed T2-weighted MRI (Figure 3(c)).

There was no evidence of infectious diseases or tumors. Given the patient's history of mild hypertension, which is a risk factor for microvascular ischemia, we diagnosed him with headache attributed to ischaemic ocular motor nerve palsy according to ICHD-3. We considered the existence of inflammation around the orbital lesion to be due to the high-intensity ring appearance on MRI. Therefore, prednisolone was administered, in a manner similar to the management of THS. Steroid pulse therapy (intravenous methylprednisolone 1000 mg/day for 3 days) was initiated, and his headache improved immediately on day two of therapy. As ptosis and diplopia persisted, steroid pulse therapy was three times the duration. These symptoms gradually improved, and he was discharged three weeks after admission with no prescription for oral prednisolone. He fully recovered six months after the onset, with no recurrence of symptoms for over 10 months.

## 4. Results

All patients had unilateral orbital pain and oculomotor nerve palsy, and two patients also showed cranial nerve VI palsy. They had no visual field defects or visual loss upon examinations by the ophthalmologist.

All patients underwent MRI examinations during the acute stage. MRI findings are summarized in Table 1. One patient was diagnosed with THS based on the appearance of granulomatous inflammation of the cavernous sinus on MRI. The other three patients had no abnormal lesions of the cavernous sinus and were diagnosed with headache attributed to ischaemic ocular motor nerve palsy due to the existence of risk factors for microvascular ischemia based on ICHD-3. All patients' MRIs revealed high-intensity ring appearance around the optic nerve of the ipsilateral lesion in brain MRI.

All patients were treated with prednisolone and responded well, particularly in terms of immediate headache improvement. Follow-up MRI was available for only one patient, which indicated the disappearance of the high-intensity ring appearance around the optic nerve. None of the patients experienced relapse of orbital pain and ophthalmoplegia after discounting prednisolone.

## 5. Discussion

Painful ophthalmoplegia is classified under three groups according to ICHD-3: THS, headache attributed to ischaemic ocular motor nerve palsy, and recurrent painful ophthalmoplegic neuropathy. THS is a granulomatous inflammatory disease of the cavernous sinus, superior orbital fissure, or the orbit [4, 5] and has been defined as unilateral orbit or periorbital pain associated with palsies of one or more of the third, fourth, and/or sixth cranial nerves caused by a granulomatous inflammation [2]. Diagnosis of THS requires the presence of the granulomatous inflammation of these lesions demonstrated by either MRI or biopsy. For the treatment of THS, prednisolone is recognized as standard therapy with positive outcomes. Headache attributed to ischaemic ocular motor nerve palsy is described as unilateral frontal and/or periorbital pain and is caused by, and associated with, clinical signs of ischaemic paresis of the ipsilateral third, fourth, and/or sixth cranial nerves [2]. Patients with headache attributed to ischaemic ocular motor nerve palsy tend to have one or more vasculopathic risk factors, including diabetes, hypertension, and hypercholesterolemia [6] with no specific abnormalities on MRI [7]. Management and effective treatment of headache attributed to ischaemic ocular motor nerve palsy, despite the history of diabetes mellitus, have not been established. Recurrent painful ophthalmoplegic neuropathy, previously known as ophthalmoplegic migraine, is described by repeated attacks of one or more ocular cranial nerves (commonly the third cranial nerve) with ipsilateral headache [2, 8]. To diagnose recurrent painful ophthalmoplegic neuropathy, recurrences of the typical symptoms and exclusion of orbital, parasellar, or posterior fossa lesions are necessary [2]. Although no granulomatous inflammation was observed on MRI, it has been reported that brain MRI can detect the gadolinium enhancement or nerve thickening of cranial nerves [2]. In our study, cases 2, 3, and 4 had no typical MRI findings of THS, and no relapse of orbital pain and ophthalmoplegia; therefore, we diagnosed these patients with headache attributed to ischaemic ocular motor nerve palsy.

MRI findings are necessary for the diagnosis of painful ophthalmoplegia. Specifically, THS and recurrent painful ophthalmoplegic neuropathy are described in terms of their typical MRI abnormalities. In this study, all patients presented with the high-intensity ring appearance around the optic nerve on STIR or fat-suppressed T2-weighted MRI without presenting with visual disturbances. Although this MRI finding has been reported in a few other studies [9, 10], its clinical utility has not been evaluated in patients with painful ophthalmoplegia. The high-intensity ring appearance around the optic nerve on fat-suppressed T2-weighted MRI was also reported in patients with THS. It has been suggested that the presence of intracranial pressure elevation or peripheral circulatory insufficiency is due to compression caused by granulomatous lesions [9]. In patients with THS, optic nerve dysfunction occurs because the inflammatory lesions in the cavernous sinus involve the orbital apex and optic nerve, and extensive lesions were observed in MRI [10]. In the present study, cases 2, 3, and 4 were diagnosed with headaches attributed to ischaemic ocular motor nerve palsy according to ICHD-3 because there were no granulomatous lesions on brain MRI. However, there is no description of the high-intensity ring appearance around the optic nerve in ICHD-3.

The high-intensity ring appearance around the optic nerve is occasionally observed with all forms of inflammation or compression, which are not disease specific changes. Moreover, the unilateral high-intensity ring appearance has been described in some inflammatory disorders such as dysthyroid optic neuropathy, optic neuritis, and inflammatory optic neuropathies [11]. Therefore, we considered the high-intensity ring appearance around the optic nerve as a significant indicator for the existence of inflammation around the orbital lesion.

To investigate the etiology of the lesion, biopsy is the gold standard. However, biopsy of the lesion is challenging unless the patient complains of visual loss. We suggest that the appearance of the high-intensity ring around the optic nerve seems to be reflected in some minor inflammation without visual disturbance, and there is some possibility of visual dysfunction being caused by the progression of the disease. When the symptoms include either a high-intensity ring appearance showing ipsilateral to headache or ophthalmoplegia, clinicians should be attentive to the patient's visual function and consider the prompt initiation of treatment.

Focusing on the prognosis of painful ophthalmoplegia, the response to steroid therapy is different in each disease diagnosed by ICHD-3. Though THS and recurrent painful ophthalmoplegic neuropathy improved well with steroid therapy due to their inflammatory pathogenesis, headaches attributed to ischaemic ocular motor nerve palsy, particularly with diabetes, do not respond similarly [3]. In this report, we administered steroid therapy to all patients because of the existence of inflammation suspected by the high-intensity ring appearance. Although cases 2, 3, and 4 were diagnosed with headaches attributed to ischaemic ocular motor nerve palsy, all patients had a positive response and excellent prognosis to steroid therapy. We supposed that the high-intensity ring appearance was caused by inflammation given the improvement following steroid therapy. Therefore, the high-intensity ring appearance may be considered as a radiological hallmark for administering steroids to patients with painful ophthalmoplegia. Additional information about the high-intensity ring appearance is needed for the diagnostic criteria of painful ophthalmoplegia.

## 6. Conclusion

Despite the absence of visual disturbances in patients with painful ophthalmoplegia, attention should be paid to potential ipsilateral high-intensity ring appearance around the optic nerve on MRI. If this is detected, the existence of inflammation in the orbital apex or cavernous sinus should be suspected. Moreover, steroid therapy should be considered for improving these symptoms.

## Figures and Tables

**Table 1:** Clinical characteristics and MRI findings of our cases.

Case	Age/Sex	Diagnosis	Side	Underlying disease	Involvement of the cranial nerves	Visual disturbance	MRI findings		Response to steroid therapy	Recurrence
							Granulomatous inflammation of the cavernous	High-intensity ring appearance around the optic nerve		
1	39/M	T	Left	−	III, VI	−	+ (left)	+ (left)	+	−
2	68/F	H	Left	Diabetes mellitus	III	−	−	+ (left)	+	−
3	76/M	H	Right	Hypertension	III	−	−	+ (right)	+	−
4	67/M	H	Right	Hypertension	III, VI	−	−	+ (right)	+	−

+, present; −, absent; MRI, magnetic resonance imaging; III, oculomotor nerve; VI, abducens nerve; T, Tolosa–Hunt syndrome; H, headache attributed to ischaemic ocular motor nerve palsy.
